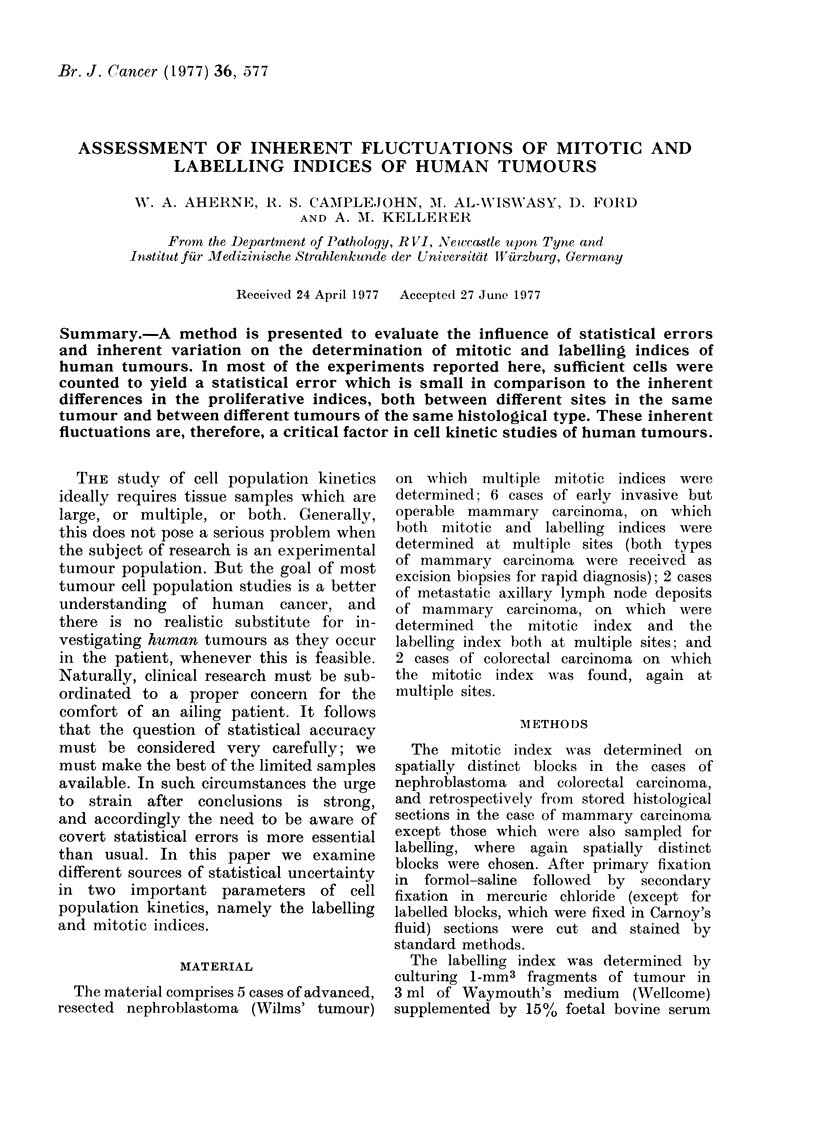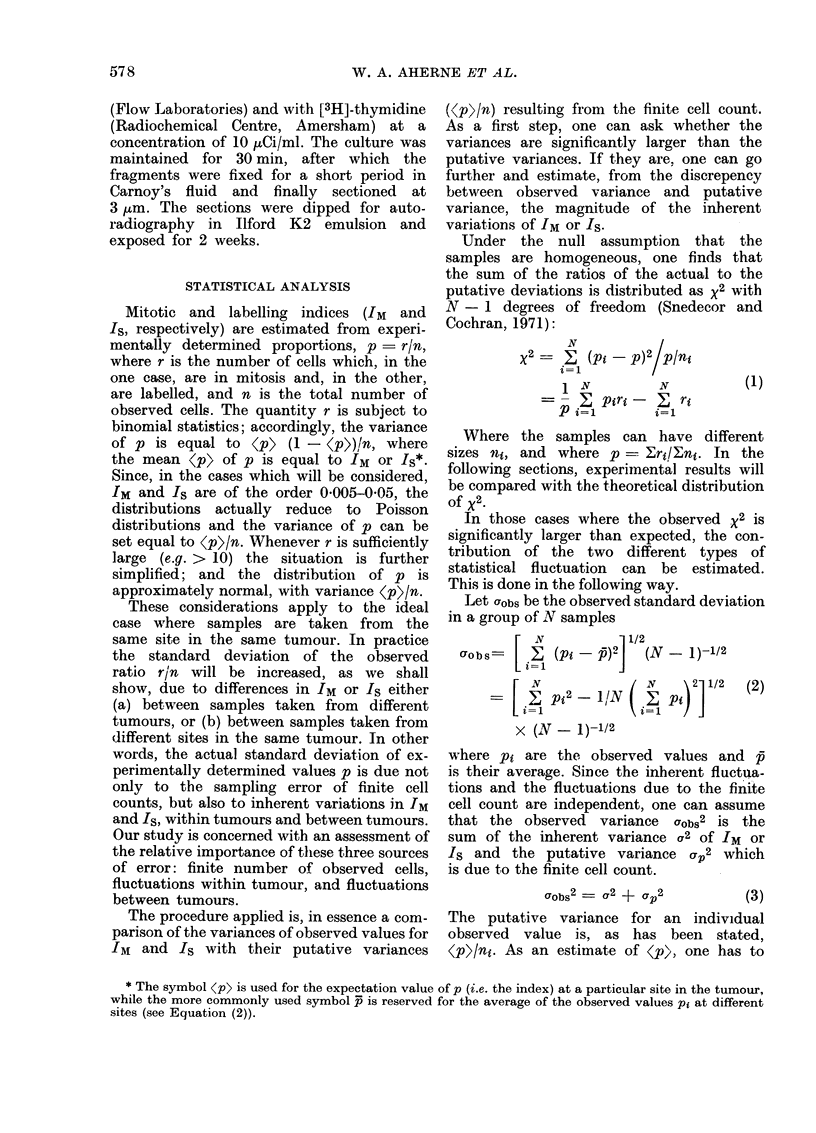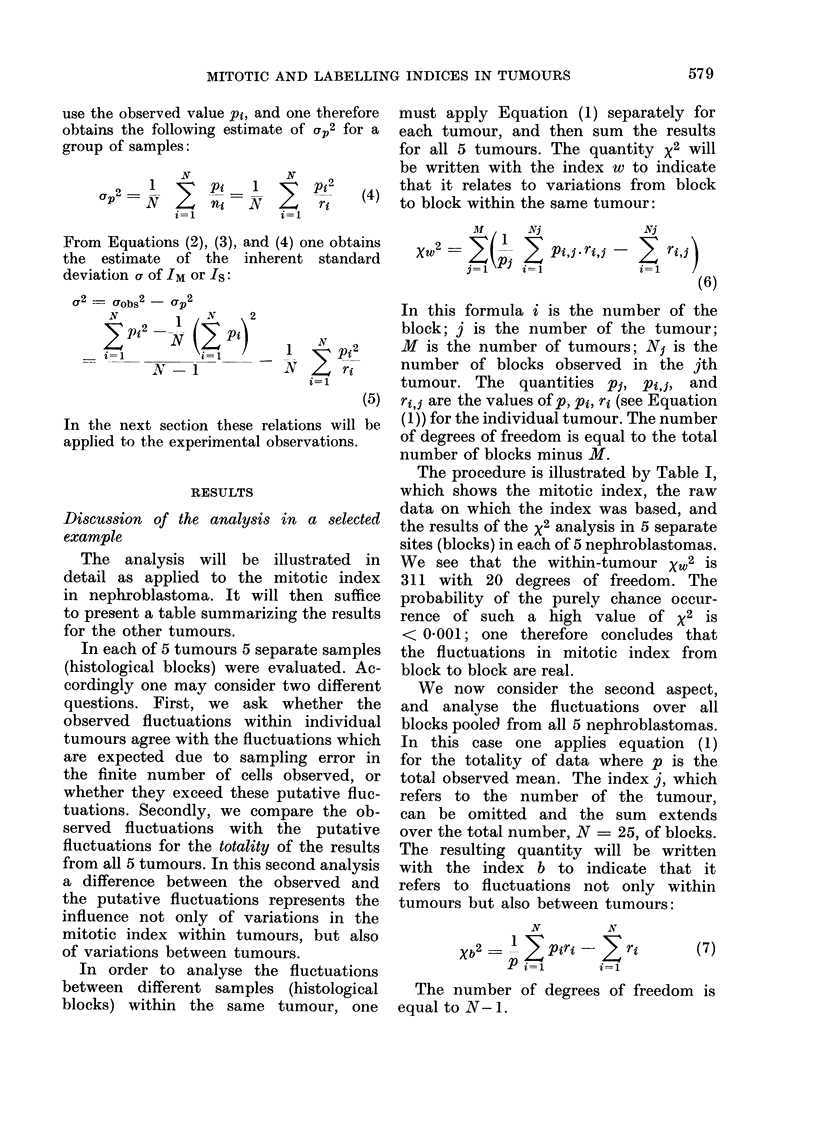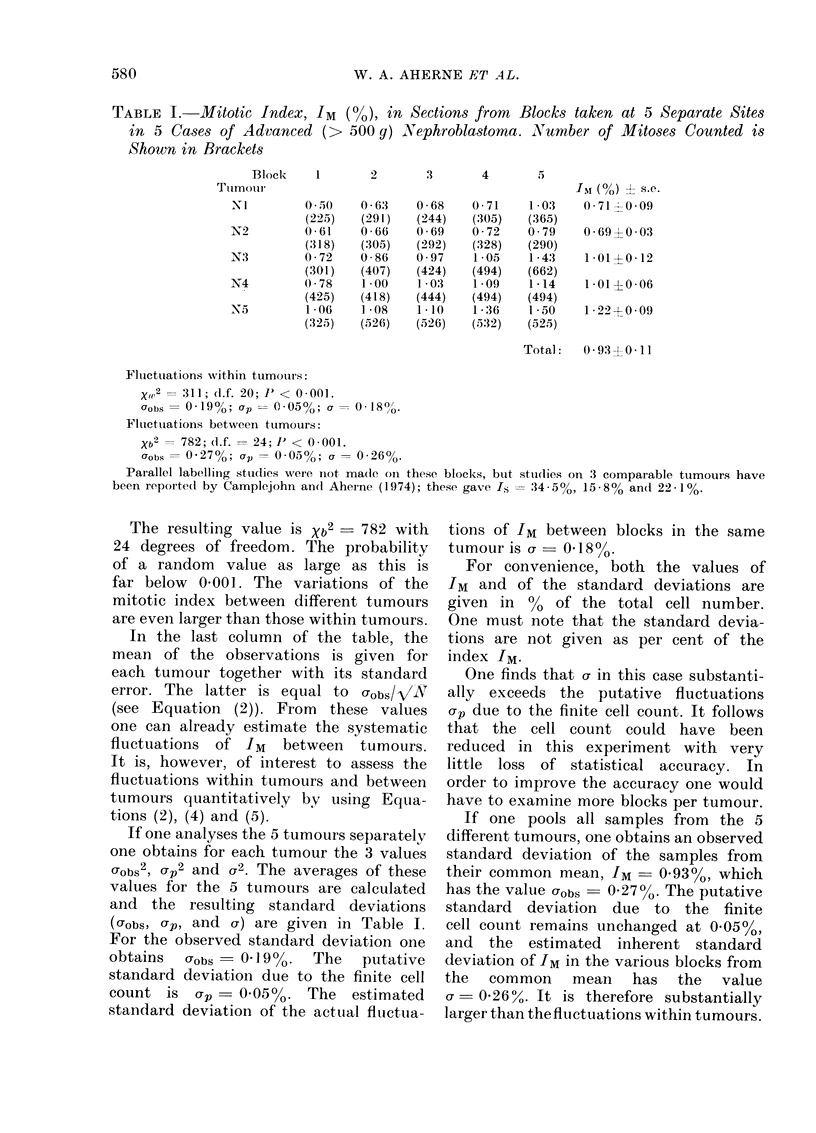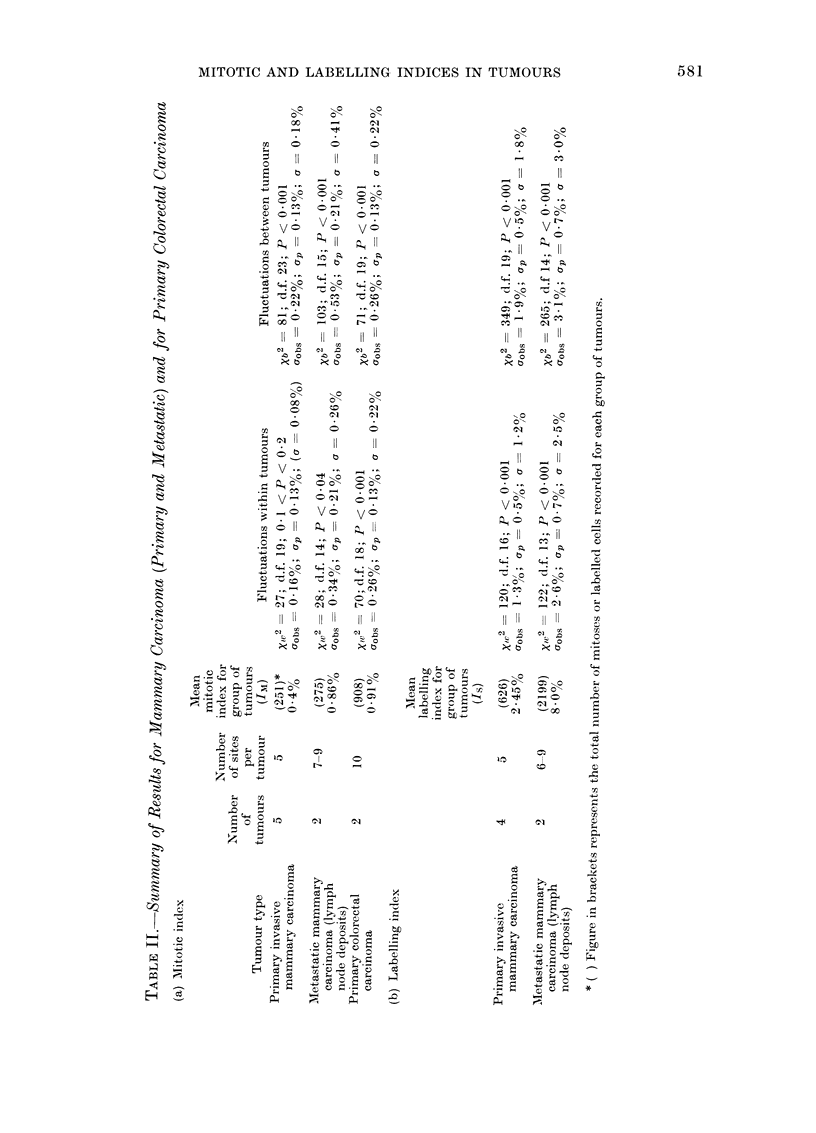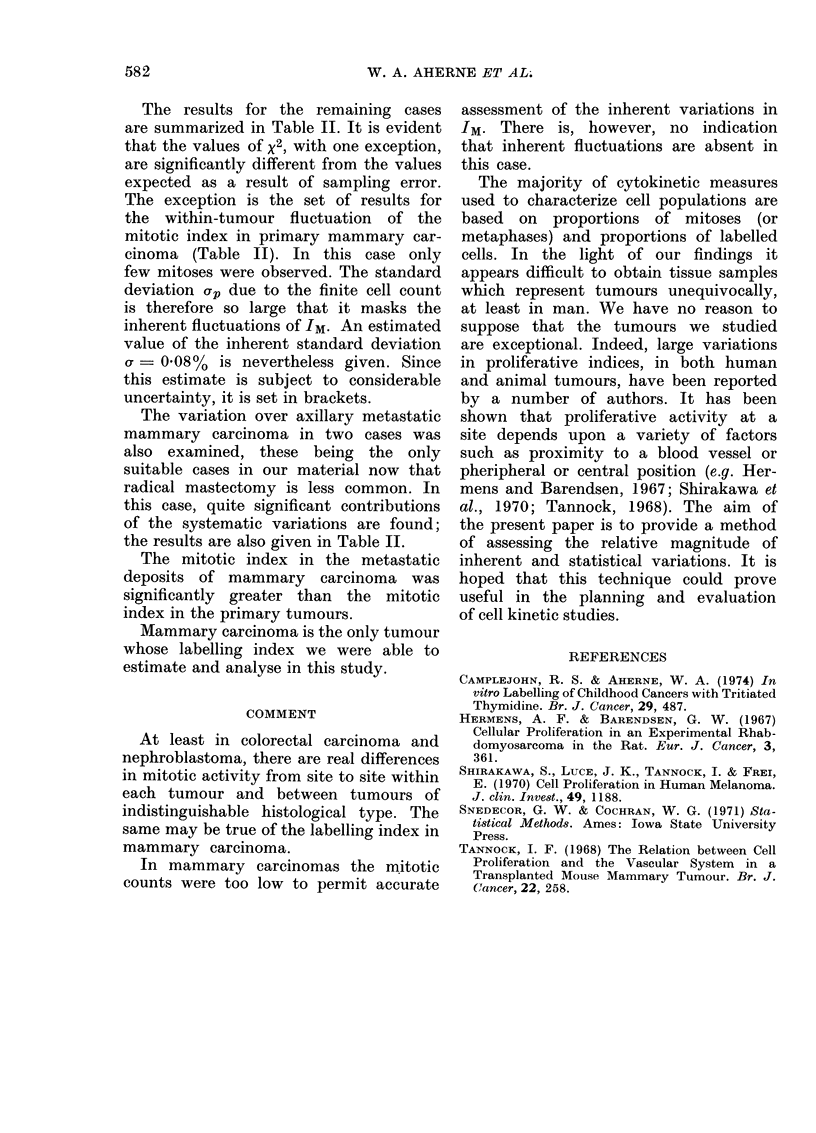# Assessment of inherent fluctuations of mitotic and labelling indices of human tumours.

**DOI:** 10.1038/bjc.1977.234

**Published:** 1977-11

**Authors:** W. A. Aherne, R. S. Camplejohn, M. Al-Wiswasy, D. Ford, A. M. Kellerer

## Abstract

A method is presented to evaluate the influence of statistical errors and inherent variation on the determination of mitotic and labelling indices of human tumours. In most of the experiments reported here, sufficient cells were counted to yield a statistical error which is small in comparison to the inherent differences in the proliferative indices, both between different sites in the same tumour and between different tumours of the same histological type. These inherent fluctuations are, theefore, a critical factor in cell kinetic studies of human tumours.


*Br. J. Cancer* (1977) **36**, 577

### ASSESSMENT OF INHERENT FLUCTUATIONS OF MITOTIC AND LABELLING INDICES OF HUMAN TUMOURS

W. A. AHERNE, R. S. CAMPLEJOHN, M. AL-WISWASY, D. FORD AND A. M. KELLERER

*From the Department of Pathology, RVI, Newcastle upon Tyne and Institut für Medizinische Strahlenkunde der Universität Würzburg, Germany*

Received 24 April 1977 Accepted 27 June 1977

**Summary.—A method is presented to evaluate the influence of statistical errors and inherent variation on the determination of mitotic and labelling indices of human tumours. In most of the experiments reported here, sufficient cells were counted to yield a statistical error which is small in comparison to the inherent differences in the proliferative indices, both between different sites in the same tumour and between different tumours of the same histological type. These inherent fluctuations are, therefore, a critical factor in cell kinetic studies of human tumours.**

THE study of cell population kinetics ideally requires tissue samples which are large, or multiple, or both. Generally, this does not pose a serious problem when the subject of research is an experimental tumour population. But the goal of most tumour cell population studies is a better understanding of human cancer, and there is no realistic substitute for investigating *human* tumours as they occur in the patient, whenever this is feasible. Naturally, clinical research must be subordinated to a proper concern for the comfort of an ailing patient. It follows that the question of statistical accuracy must be considered very carefully; we must make the best of the limited samples available. In such circumstances the urge to strain after conclusions is strong, and accordingly the need to be aware of covert statistical errors is more essential than usual. In this paper we examine different sources of statistical uncertainty in two important parameters of cell population kinetics, namely the labelling and mitotic indices.

### MATERIAL

The material comprises 5 cases of advanced, resected nephroblastoma (Wilms' tumour) on which multiple mitotic indices were determined; 6 cases of early invasive but operable mammary carcinoma, on which both mitotic and labelling indices were determined at multiple sites (both types of mammary carcinoma were received as excision biopsies for rapid diagnosis); 2 cases of metastatic axillary lymph node deposits of mammary carcinoma, on which were determined the mitotic index and the labelling index both at multiple sites; and 2 cases of colorectal carcinoma on which the mitotic index was found, again at multiple sites.

### METHODS

The mitotic index was determined on spatially distinct blocks in the cases of nephroblastoma and colorectal carcinoma, and retrospectively from stored histological sections in the case of mammary carcinoma except those which were also sampled for labelling, where again spatially distinct blocks were chosen. After primary fixation in formol–saline followed by secondary fixation in mercuric chloride (except for labelled blocks, which were fixed in Carnoy's fluid) sections were cut and stained by standard methods.

The labelling index was determined by culturing 1-$mm^3$ fragments of tumour in 3 ml of Waymouth's medium (Wellcome) supplemented by 15% foetal bovine serum

(Flow Laboratories) and with [$^3$H]-thymidine (Radiochemical Centre, Amersham) at a concentration of 10 $\mu$Ci/ml. The culture was maintained for 30 min, after which the fragments were fixed for a short period in Carnoy's fluid and finally sectioned at 3 $\mu$m. The sections were dipped for autoradiography in Ilford K2 emulsion and exposed for 2 weeks.

### STATISTICAL ANALYSIS

Mitotic and labelling indices ($I_M$ and $I_S$, respectively) are estimated from experimentally determined proportions, $p = r/n$, where $r$ is the number of cells which, in the one case, are in mitosis and, in the other, are labelled, and $n$ is the total number of observed cells. The quantity $r$ is subject to binomial statistics; accordingly, the variance of $p$ is equal to $\langle p \rangle$ $(1 - \langle p \rangle)/n$, where the mean $\langle p \rangle$ of $p$ is equal to $I_M$ or $I_S$*. Since, in the cases which will be considered, $I_M$ and $I_S$ are of the order 0·005–0·05, the distributions actually reduce to Poisson distributions and the variance of $p$ can be set equal to $\langle p \rangle/n$. Whenever $r$ is sufficiently large (*e.g.* $> 10$) the situation is further simplified; and the distribution of $p$ is approximately normal, with variance $\langle p \rangle/n$.

These considerations apply to the ideal case where samples are taken from the same site in the same tumour. In practice the standard deviation of the observed ratio $r/n$ will be increased, as we shall show, due to differences in $I_M$ or $I_S$ either (a) between samples taken from different tumours, or (b) between samples taken from different sites in the same tumour. In other words, the actual standard deviation of experimentally determined values $p$ is due not only to the sampling error of finite cell counts, but also to inherent variations in $I_M$ and $I_S$, within tumours and between tumours. Our study is concerned with an assessment of the relative importance of these three sources of error: finite number of observed cells, fluctuations within tumour, and fluctuations between tumours.

The procedure applied is, in essence a comparison of the variances of observed values for $I_M$ and $I_S$ with their putative variances ($\langle p \rangle/n$) resulting from the finite cell count. As a first step, one can ask whether the variances are significantly larger than the putative variances. If they are, one can go further and estimate, from the discrepency between observed variance and putative variance, the magnitude of the inherent variations of $I_M$ or $I_S$.

Under the null assumption that the samples are homogeneous, one finds that the sum of the ratios of the actual to the putative deviations is distributed as $\chi^2$ with $N - 1$ degrees of freedom (Snedecor and Cochran, 1971):

$$\chi^2 = \sum_{i=1}^{N} (p_i - p)^2 \Big/ p/n_i = \frac{1}{p}\sum_{i=1}^{N} p_i r_i - \sum_{i=1}^{N} r_i \tag{1}$$

Where the samples can have different sizes $n_i$, and where $p = \Sigma r_i/\Sigma n_i$. In the following sections, experimental results will be compared with the theoretical distribution of $\chi^2$.

In those cases where the observed $\chi^2$ is significantly larger than expected, the contribution of the two different types of statistical fluctuation can be estimated. This is done in the following way.

Let $\sigma_{obs}$ be the observed standard deviation in a group of $N$ samples

$$\sigma_{obs} = \left[\sum_{i=1}^{N} (p_i - \bar{p})^2\right]^{1/2} (N-1)^{-1/2} = \left[\sum_{i=1}^{N} p_i^2 - 1/N\left(\sum_{i=1}^{N} p_i\right)^2\right]^{1/2} \times (N-1)^{-1/2} \tag{2}$$

where $p_i$ are the observed values and $\bar{p}$ is their average. Since the inherent fluctuations and the fluctuations due to the finite cell count are independent, one can assume that the observed variance $\sigma_{obs}^2$ is the sum of the inherent variance $\sigma^2$ of $I_M$ or $I_S$ and the putative variance $\sigma_p^2$ which is due to the finite cell count.

$$\sigma_{obs}^2 = \sigma^2 + \sigma_p^2 \tag{3}$$

The putative variance for an individual observed value is, as has been stated, $\langle p \rangle/n_i$. As an estimate of $\langle p \rangle$, one has to

* The symbol $\langle p \rangle$ is used for the expectation value of $p$ (*i.e.* the index) at a particular site in the tumour, while the more commonly used symbol $\bar{p}$ is reserved for the average of the observed values $p_i$ at different sites (see Equation (2)).

use the observed value $p_i$, and one therefore obtains the following estimate of $\sigma_p{}^2$ for a group of samples:

$$\sigma_p{}^2 = \frac{1}{N} \sum_{i=1}^{N} \frac{p_i}{n_i} = \frac{1}{N} \sum_{i=1}^{N} \frac{p_i{}^2}{r_i} \quad (4)$$

From Equations (2), (3), and (4) one obtains the estimate of the inherent standard deviation $\sigma$ of $I_M$ or $I_S$:

$$\sigma^2 = \sigma_{obs}{}^2 - \sigma_p{}^2 = \frac{\sum_{i=1}^{N} p_i{}^2 - \frac{1}{N}\left(\sum_{i=1}^{N} p_i\right)^2}{N-1} - \frac{1}{N}\sum_{i=1}^{N} \frac{p_i{}^2}{r_i} \quad (5)$$

In the next section these relations will be applied to the experimental observations.

### RESULTS

### *Discussion of the analysis in a selected example*

The analysis will be illustrated in detail as applied to the mitotic index in nephroblastoma. It will then suffice to present a table summarizing the results for the other tumours.

In each of 5 tumours 5 separate samples (histological blocks) were evaluated. Accordingly one may consider two different questions. First, we ask whether the observed fluctuations within individual tumours agree with the fluctuations which are expected due to sampling error in the finite number of cells observed, or whether they exceed these putative fluctuations. Secondly, we compare the observed fluctuations with the putative fluctuations for the *totality* of the results from all 5 tumours. In this second analysis a difference between the observed and the putative fluctuations represents the influence not only of variations in the mitotic index within tumours, but also of variations between tumours.

In order to analyse the fluctuations between different samples (histological blocks) within the same tumour, one must apply Equation (1) separately for each tumour, and then sum the results for all 5 tumours. The quantity $\chi^2$ will be written with the index $w$ to indicate that it relates to variations from block to block within the same tumour:

$$\chi_w{}^2 = \sum_{j=1}^{M}\left(\frac{1}{p_j}\sum_{i=1}^{N_j} p_{i,j}.r_{i,j} - \sum_{i=1}^{N_j} r_{i,j}\right) \quad (6)$$

In this formula $i$ is the number of the block; $j$ is the number of the tumour; $M$ is the number of tumours; $N_j$ is the number of blocks observed in the $j$th tumour. The quantities $p_j$, $p_{i,j}$, and $r_{i,j}$ are the values of $p$, $p_i$, $r_i$ (see Equation (1)) for the individual tumour. The number of degrees of freedom is equal to the total number of blocks minus $M$.

The procedure is illustrated by Table I, which shows the mitotic index, the raw data on which the index was based, and the results of the $\chi^2$ analysis in 5 separate sites (blocks) in each of 5 nephroblastomas. We see that the within-tumour $\chi_w{}^2$ is 311 with 20 degrees of freedom. The probability of the purely chance occurrence of such a high value of $\chi^2$ is $< 0{\cdot}001$; one therefore concludes that the fluctuations in mitotic index from block to block are real.

We now consider the second aspect, and analyse the fluctuations over all blocks pooled from all 5 nephroblastomas. In this case one applies equation (1) for the totality of data where $p$ is the total observed mean. The index $j$, which refers to the number of the tumour, can be omitted and the sum extends over the total number, $N = 25$, of blocks. The resulting quantity will be written with the index $b$ to indicate that it refers to fluctuations not only within tumours but also between tumours:

$$\chi_b{}^2 = \frac{1}{p}\sum_{i=1}^{N} p_i r_i - \sum_{i=1}^{N} r_i \quad (7)$$

The number of degrees of freedom is equal to $N-1$.

TABLE I.—*Mitotic Index, $I_M$ (%), in Sections from Blocks taken at 5 Separate Sites in 5 Cases of Advanced (> 500 g) Nephroblastoma. Number of Mitoses Counted is Shown in Brackets*

| Block / Tumour | 1 | 2 | 3 | 4 | 5 | $I_M$ (%) ± s.e. |
|---|---|---|---|---|---|---|
| N1 | 0·50 (225) | 0·63 (291) | 0·68 (244) | 0·71 (305) | 1·03 (365) | 0·71±0·09 |
| N2 | 0·61 (318) | 0·66 (305) | 0·69 (292) | 0·72 (328) | 0·79 (290) | 0·69±0·03 |
| N3 | 0·72 (301) | 0·86 (407) | 0·97 (424) | 1·05 (494) | 1·43 (662) | 1·01±0·12 |
| N4 | 0·78 (425) | 1·00 (418) | 1·03 (444) | 1·09 (494) | 1·14 (494) | 1·01±0·06 |
| N5 | 1·06 (325) | 1·08 (526) | 1·10 (526) | 1·36 (532) | 1·50 (525) | 1·22±0·09 |
| | | | | | Total: | 0·93±0·11 |

Fluctuations within tumours:

$\chi_w^2 = 311$; d.f. 20; $P < 0{\cdot}001$.
$\sigma_{obs} = 0{\cdot}19\%$; $\sigma_p = 0{\cdot}05\%$; $\sigma = 0{\cdot}18\%$.

Fluctuations between tumours:

$\chi_b^2 = 782$; d.f. $= 24$; $P < 0{\cdot}001$.
$\sigma_{obs} = 0{\cdot}27\%$; $\sigma_p = 0{\cdot}05\%$; $\sigma = 0{\cdot}26\%$.

Parallel labelling studies were not made on these blocks, but studies on 3 comparable tumours have been reported by Camplejohn and Aherne (1974); these gave $I_S = 34{\cdot}5\%$, $15{\cdot}8\%$ and $22{\cdot}1\%$.

The resulting value is $\chi_b^2 = 782$ with 24 degrees of freedom. The probability of a random value as large as this is far below 0·001. The variations of the mitotic index between different tumours are even larger than those within tumours.

In the last column of the table, the mean of the observations is given for each tumour together with its standard error. The latter is equal to $\sigma_{obs}/\sqrt{N}$ (see Equation (2)). From these values one can already estimate the systematic fluctuations of $I_M$ between tumours. It is, however, of interest to assess the fluctuations within tumours and between tumours quantitatively by using Equations (2), (4) and (5).

If one analyses the 5 tumours separately one obtains for each tumour the 3 values $\sigma_{obs}^2$, $\sigma_p^2$ and $\sigma^2$. The averages of these values for the 5 tumours are calculated and the resulting standard deviations ($\sigma_{obs}$, $\sigma_p$, and $\sigma$) are given in Table I. For the observed standard deviation one obtains $\sigma_{obs} = 0{\cdot}19\%$. The putative standard deviation due to the finite cell count is $\sigma_p = 0{\cdot}05\%$. The estimated standard deviation of the actual fluctuations of $I_M$ between blocks in the same tumour is $\sigma = 0{\cdot}18\%$.

For convenience, both the values of $I_M$ and of the standard deviations are given in % of the total cell number. One must note that the standard deviations are not given as per cent of the index $I_M$.

One finds that $\sigma$ in this case substantially exceeds the putative fluctuations $\sigma_p$ due to the finite cell count. It follows that the cell count could have been reduced in this experiment with very little loss of statistical accuracy. In order to improve the accuracy one would have to examine more blocks per tumour.

If one pools all samples from the 5 different tumours, one obtains an observed standard deviation of the samples from their common mean, $I_M = 0{\cdot}93\%$, which has the value $\sigma_{obs} = 0{\cdot}27\%$. The putative standard deviation due to the finite cell count remains unchanged at 0·05%, and the estimated inherent standard deviation of $I_M$ in the various blocks from the common mean has the value $\sigma = 0{\cdot}26\%$. It is therefore substantially larger than the fluctuations within tumours.

TABLE II.—*Summary of Results for Mammary Carcinoma (Primary and Metastatic) and for Primary Colorectal Carcinoma*

(a) Mitotic index

| Tumour type | Number of tumours | Number of sites per tumour | Mean mitotic index for group of tumours ($I_M$) | Fluctuations within tumours | Fluctuations between tumours |
|---|---|---|---|---|---|
| Primary invasive mammary carcinoma | 5 | 5 | (251)* 0·4% | $\chi_w^2 = 27$; d.f. 19; $0 \cdot 1 < P < 0 \cdot 2$<br>$\sigma_{obs} = 0 \cdot 16\%$; $\sigma_p = 0 \cdot 13\%$; ($\sigma = 0 \cdot 08\%$) | $\chi_b^2 = 81$; d.f. 23; $P < 0 \cdot 001$<br>$\sigma_{obs} = 0 \cdot 22\%$; $\sigma_p = 0 \cdot 13\%$; $\sigma = 0 \cdot 18\%$ |
| Metastatic mammary carcinoma (lymph node deposits) | 2 | 7–9 | (275) 0·86% | $\chi_w^2 = 28$; d.f. 14; $P < 0 \cdot 04$<br>$\sigma_{obs} = 0 \cdot 34\%$; $\sigma_p = 0 \cdot 21\%$; $\sigma = 0 \cdot 26\%$ | $\chi_b^2 = 103$; d.f. 15; $P < 0 \cdot 001$<br>$\sigma_{obs} = 0 \cdot 53\%$; $\sigma_p = 0 \cdot 21\%$; $\sigma = 0 \cdot 41\%$ |
| Primary colorectal carcinoma | 2 | 10 | (908) 0·91% | $\chi_w^2 = 70$; d.f. 18; $P < 0 \cdot 001$<br>$\sigma_{obs} = 0 \cdot 26\%$; $\sigma_p = 0 \cdot 13\%$; $\sigma = 0 \cdot 22\%$ | $\chi_b^2 = 71$; d.f. 19; $P < 0 \cdot 001$<br>$\sigma_{obs} = 0 \cdot 26\%$; $\sigma_p = 0 \cdot 13\%$; $\sigma = 0 \cdot 22\%$ |

(b) Labelling index

| Tumour type | Number of tumours | Number of sites per tumour | Mean labelling index for group of tumours ($I_S$) | Fluctuations within tumours | Fluctuations between tumours |
|---|---|---|---|---|---|
| Primary invasive mammary carcinoma | 4 | 5 | (626) 2·45% | $\chi_w^2 = 120$; d.f. 16; $P < 0 \cdot 001$<br>$\sigma_{obs} = 1 \cdot 3\%$; $\sigma_p = 0 \cdot 5\%$; $\sigma = 1 \cdot 2\%$ | $\chi_b^2 = 349$; d.f. 19; $P < 0 \cdot 001$<br>$\sigma_{obs} = 1 \cdot 9\%$; $\sigma_p = 0 \cdot 5\%$; $\sigma = 1 \cdot 8\%$ |
| Metastatic mammary carcinoma (lymph node deposits) | 2 | 6–9 | (2199) 8·0% | $\chi_w^2 = 122$; d.f. 13; $P < 0 \cdot 001$<br>$\sigma_{obs} = 2 \cdot 6\%$; $\sigma_p = 0 \cdot 7\%$; $\sigma = 2 \cdot 5\%$ | $\chi_b^2 = 265$; d.f 14; $P < 0 \cdot 001$<br>$\sigma_{obs} = 3 \cdot 1\%$; $\sigma_p = 0 \cdot 7\%$; $\sigma = 3 \cdot 0\%$ |

* ( ) Figure in brackets represents the total number of mitoses or labelled cells recorded for each group of tumours.

The results for the remaining cases are summarized in Table II. It is evident that the values of $\chi^2$, with one exception, are significantly different from the values expected as a result of sampling error. The exception is the set of results for the within-tumour fluctuation of the mitotic index in primary mammary carcinoma (Table II). In this case only few mitoses were observed. The standard deviation $\sigma_p$ due to the finite cell count is therefore so large that it masks the inherent fluctuations of $I_M$. An estimated value of the inherent standard deviation $\sigma = 0{\cdot}08\%$ is nevertheless given. Since this estimate is subject to considerable uncertainty, it is set in brackets.

The variation over axillary metastatic mammary carcinoma in two cases was also examined, these being the only suitable cases in our material now that radical mastectomy is less common. In this case, quite significant contributions of the systematic variations are found; the results are also given in Table II.

The mitotic index in the metastatic deposits of mammary carcinoma was significantly greater than the mitotic index in the primary tumours.

Mammary carcinoma is the only tumour whose labelling index we were able to estimate and analyse in this study.

### COMMENT

At least in colorectal carcinoma and nephroblastoma, there are real differences in mitotic activity from site to site within each tumour and between tumours of indistinguishable histological type. The same may be true of the labelling index in mammary carcinoma.

In mammary carcinomas the mitotic counts were too low to permit accurate assessment of the inherent variations in $I_M$. There is, however, no indication that inherent fluctuations are absent in this case.

The majority of cytokinetic measures used to characterize cell populations are based on proportions of mitoses (or metaphases) and proportions of labelled cells. In the light of our findings it appears difficult to obtain tissue samples which represent tumours unequivocally, at least in man. We have no reason to suppose that the tumours we studied are exceptional. Indeed, large variations in proliferative indices, in both human and animal tumours, have been reported by a number of authors. It has been shown that proliferative activity at a site depends upon a variety of factors such as proximity to a blood vessel or pheripheral or central position (*e.g.* Hermens and Barendsen, 1967; Shirakawa *et al.*, 1970; Tannock, 1968). The aim of the present paper is to provide a method of assessing the relative magnitude of inherent and statistical variations. It is hoped that this technique could prove useful in the planning and evaluation of cell kinetic studies.

### REFERENCES

CAMPLEJOHN, R. S. & AHERNE, W. A. (1974) *In vitro* Labelling of Childhood Cancers with Tritiated Thymidine. *Br. J. Cancer*, **29**, 487.

HERMENS, A. F. & BARENDSEN, G. W. (1967) Cellular Proliferation in an Experimental Rhabdomyosarcoma in the Rat. *Eur. J. Cancer*, **3**, 361.

SHIRAKAWA, S., LUCE, J. K., TANNOCK, I. & FREI, E. (1970) Cell Proliferation in Human Melanoma. *J. clin. Invest.*, **49**, 1188.

SNEDECOR, G. W. & COCHRAN, W. G. (1971) *Statistical Methods*. Ames: Iowa State University Press.

TANNOCK, I. F. (1968) The Relation between Cell Proliferation and the Vascular System in a Transplanted Mouse Mammary Tumour. *Br. J. Cancer*, **22**, 258.